# 9,10-Dioxo-9,10-di­hydro­anthracene-1,4-diyl di­acetate

**DOI:** 10.1107/S1600536813010635

**Published:** 2013-04-27

**Authors:** Jing-Jing Zhang, Cai-Xia Yin, Fang-Jun Huo

**Affiliations:** a Institute of Molecular Science, Chemical Biology and Molecular Engineering Laboratory of Education Ministry, University of Shanxi, Taiyuan, Shanxi 030006, People’s Republic of China; b Research Institute of Applied Chemistry, University of Shanxi, Taiyuan, Shanxi 030006, People’s Republic of China

## Abstract

In the title compound, C_18_H_12_O_6_, the anthra­quinone ring system is nearly planar [maximum deviation = 0.161 (3) Å] and both acetate groups are located on the same side of the ring plane. A supra­molecular architecture arises in the crystal owing to π–π stacking between parallel benzene rings of adjacent mol­ecules [centroid–centroid distance = 3.883 (4) Å] and weak inter­molecular C—H⋯O hydrogen bonding.

## Related literature

For applications of the title compound, see: Mal *et al.* (2007 ▶). For related compounds, see: Gianneschi *et al.* (2005 ▶); Thomas (2007 ▶); Lee & Lin (2008 ▶); Han *et al.* (2009 ▶, 2010 ▶); Lusby (2012 ▶).
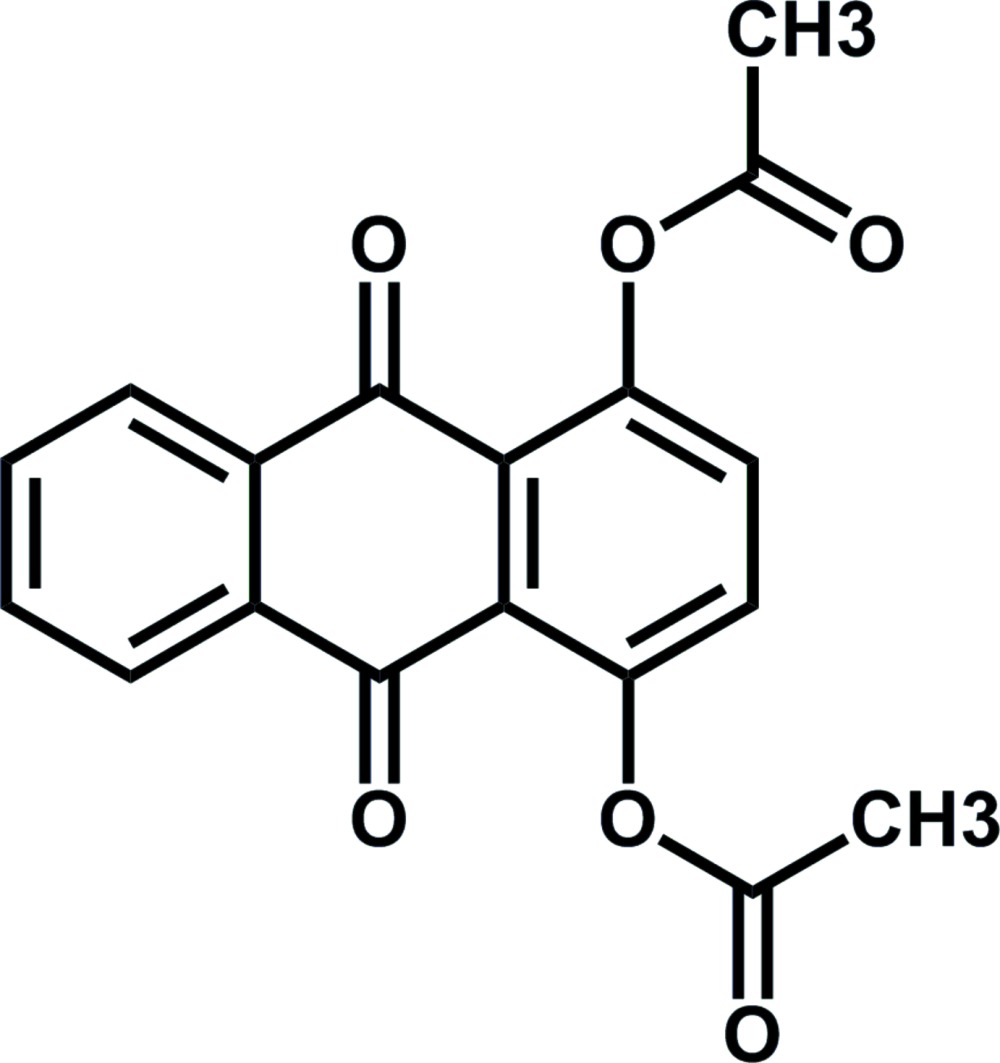


## Experimental

### 

#### Crystal data

C_18_H_12_O_6_
*M* *_r_* = 324.28
Triclinic, 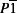

*a* = 8.208 (7) Å
*b* = 9.730 (8) Å
*c* = 9.902 (8) Å
α = 73.257 (16)°
β = 79.986 (14)°
γ = 80.770 (14)°
*V* = 740.7 (10) Å^3^
*Z* = 2
Mo *K*α radiation
μ = 0.11 mm^−1^
*T* = 296 K
0.20 × 0.15 × 0.12 mm

#### Data collection

Bruker SMART 1000 CCD area-detector diffractometer
Absorption correction: multi-scan (*SADABS*; Bruker, 2001 ▶) *T*_min_ = 0.978, *T*_max_ = 0.987
4006 measured reflections
2610 independent reflections
1616 reflections with *I* > 2σ(*I*)
*R*_int_ = 0.023

#### Refinement

*R*[*F* ^2^ > 2σ(*F* ^2^)] = 0.049
*wR*(*F* ^2^) = 0.152
*S* = 1.01
2610 reflections
219 parameters
H-atom parameters constrained
Δρ_max_ = 0.25 e Å^−3^
Δρ_min_ = −0.20 e Å^−3^

### 

Data collection: *SMART* (Bruker, 2007 ▶); cell refinement: *SAINT* (Bruker, 2007 ▶); data reduction: *SAINT*; program(s) used to solve structure: *SHELXS97* (Sheldrick, 2008 ▶); program(s) used to refine structure: *SHELXL97* (Sheldrick, 2008 ▶); molecular graphics: *SHELXTL* (Sheldrick, 2008 ▶); software used to prepare material for publication: *SHELXTL*.

## Supplementary Material

Crystal structure: contains datablock(s) I, global. DOI: 10.1107/S1600536813010635/xu5688sup1.cif

Structure factors: contains datablock(s) I. DOI: 10.1107/S1600536813010635/xu5688Isup2.hkl

Supplementary material file. DOI: 10.1107/S1600536813010635/xu5688Isup3.cml

Additional supplementary materials:  crystallographic information; 3D view; checkCIF report

## Figures and Tables

**Table 1:** Hydrogen-bond geometry (Å, °)

*D*—H⋯*A*	*D*—H	H⋯*A*	*D*⋯*A*	*D*—H⋯*A*
C18—H18*A*⋯O2^i^	0.96	2.51	3.425 (4)	159

Symmetry code: (i) 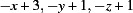.

**Fig. 1.**
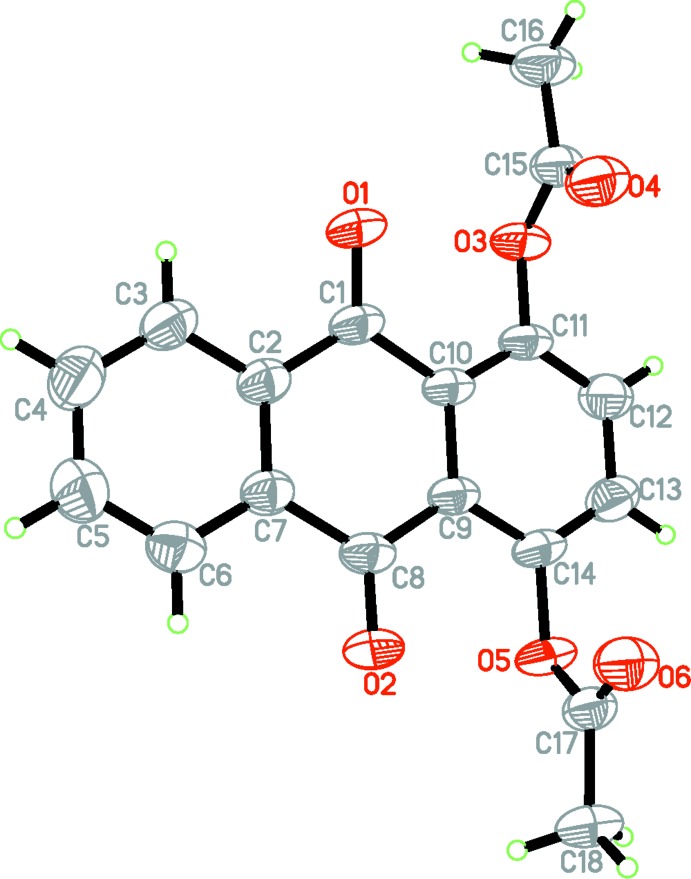
A view of the molecular structure of (I) with the atom-numbering scheme.

## supplementary crystallographic information

### Comment

The title compound has symmetry space structure and obvious color. It can be used to synthesize various dyes and are common structural subunits of many biologically active quinonoids (Mal *et al.*, 2007). It also can be modified into synthetic dyes intermediates, 1,4-diamino anthraquinone. Its readily deprotection of acetate groups forms 1,4-dihydroxyanthraquinone (1,4-DHA), which can be induced to self-assembly to form a metallo-supramolecular coordination polymers under certain condition (Gianneschi *et al.*, 2005; Thomas, 2007; Lee & Lin, 2008) and demonstrate good selectivity and binding for planar aromatic guests, small organic molecules and transitional metal ions, such as dichloromethane and iridium (Han *et al.*, 2009; Lusby, 2012; Han *et al.*, 2010)

The molecular conformation is illustrated in Fig. 1. In the title compound, C_18_H_12_O_6_, the anthraquinone ring system is nearly planar [the maximum deviation being 0.161 (3) Å], both acetate groups are located on the same side of the ring plane. A three-dimensional supramolecular architecture arises in the crystal owing to π-π stacking between centro-symmetrically related benzene rings [centroid-centroid distance 3.883 (4) Å] and weak intermolecular C—H···O hydrogen bondig.

### Experimental

To a stirred solution of 1,4-dihydrory-9,10-anthraquinone (4.6 g, 19.1 mmoL) in CH_2_Cl_2_ (50 ml), Ac_2_O (2 ml) and pyridine (one drop) were added. After the solution was stirred overnight at room temperature, it was evaporated under vacuum. The crude products were dissolved in water and then extracted with EtOAc. The combinded organic layer was washed with brine, and then dried with Na_2_SO_4_. The solvent was removed under the reduced pressure and the residue was purified by column chromatography using petroleum ether/ethyl acetate (*v*/*v* 2:1, *R*_f_ = 0.50) as an eluent to afford 9,10-dioxo-9,10-dihydroanthracene-1,4-diyl diacetate as a white solid. Colorless single crystals were obtained from the ethyl acetate solution.

### Refinement

All H atoms were initially lacated in a difference Fourier map. H atoms on C*sp*^3^ were treated as riding with C—H = 0.96 Å and *U*_iso_(H) = 1.5*U*_eq_(C) of the parent atom. The H atoms on C*sp*^2^ were treated as riding with C—H = 0.93 Å and *U*_iso_(H) = 1.2*U*_eq_(C).

### Crystal data

**Table:**

C_18_H_12_O_6_	*Z* = 2
*M**_r_* = 324.28	*F*(000) = 336
Triclinic, *P*1	*D*_x_ = 1.454 Mg m^−^^3^
Hall symbol: -P 1	Mo *K*α radiation, λ = 0.71073 Å
*a* = 8.208 (7) Å	Cell parameters from 1029 reflections
*b* = 9.730 (8) Å	θ = 2.5–25.9°
*c* = 9.902 (8) Å	µ = 0.11 mm^−^^1^
α = 73.257 (16)°	*T* = 296 K
β = 79.986 (14)°	Block, colorless
γ = 80.770 (14)°	0.20 × 0.15 × 0.12 mm
*V* = 740.7 (10) Å^3^	

### Data collection

**Table:**

Bruker SMART 1000 CCD area-detector diffractometer	2610 independent reflections
Radiation source: fine-focus sealed tube	1616 reflections with *I* > 2σ(*I*)
Graphite monochromator	*R*_int_ = 0.023
ω scans	θ_max_ = 25.1°, θ_min_ = 2.2°
Absorption correction: multi-scan (*SADABS*; Bruker, 2001)	*h* = −9→9
*T*_min_ = 0.978, *T*_max_ = 0.987	*k* = −11→9
4006 measured reflections	*l* = −8→11

### Refinement

**Table:**

Refinement on *F*^2^	Primary atom site location: structure-invariant direct methods
Least-squares matrix: full	Secondary atom site location: difference Fourier map
*R*[*F*^2^ > 2σ(*F*^2^)] = 0.049	Hydrogen site location: inferred from neighbouring sites
*wR*(*F*^2^) = 0.152	H-atom parameters constrained
*S* = 1.01	*w* = 1/[σ^2^(*F*_o_^2^) + (0.0852*P*)^2^] where *P* = (*F*_o_^2^ + 2*F*_c_^2^)/3
2610 reflections	(Δ/σ)_max_ < 0.001
219 parameters	Δρ_max_ = 0.25 e Å^−^^3^
0 restraints	Δρ_min_ = −0.20 e Å^−^^3^

### Special details

**Table:**

Geometry. All e.s.d.'s (except the e.s.d. in the dihedral angle between two l.s. planes) are estimated using the full covariance matrix. The cell e.s.d.'s are taken into account individually in the estimation of e.s.d.'s in distances, angles and torsion angles; correlations between e.s.d.'s in cell parameters are only used when they are defined by crystal symmetry. An approximate (isotropic) treatment of cell e.s.d.'s is used for estimating e.s.d.'s involving l.s. planes.
Refinement. Refinement of *F*^2^ against ALL reflections. The weighted *R*-factor *wR* and goodness of fit *S* are based on *F*^2^, conventional *R*-factors *R* are based on *F*, with *F* set to zero for negative *F*^2^. The threshold expression of *F*^2^ > 2 σ (*F*^2^) is used only for calculating *R*-factors (gt) *etc*. and is not relevant to the choice of reflections for refinement. *R*-factors based on *F*^2^ are statistically about twice as large as those based on *F*, and *R*-factors based on ALL data will be even larger.

### Fractional atomic coordinates and isotropic or equivalent isotropic displacement parameters (Å^2^)

**Table:**

	*x*	*y*	*z*	*U*_iso_*/*U*_eq_	
O1	0.6269 (2)	0.7129 (2)	0.92996 (18)	0.0591 (6)	
O2	1.1612 (2)	0.4505 (2)	0.66625 (19)	0.0548 (5)	
O3	0.5808 (2)	0.92058 (19)	0.69178 (18)	0.0484 (5)	
O4	0.7230 (2)	1.0267 (2)	0.80172 (19)	0.0571 (5)	
O5	1.1375 (2)	0.6552 (2)	0.42121 (16)	0.0470 (5)	
O6	1.3324 (2)	0.7313 (2)	0.50884 (19)	0.0557 (5)	
C1	0.7353 (3)	0.6484 (3)	0.8615 (2)	0.0402 (6)	
C2	0.8032 (3)	0.4978 (3)	0.9257 (2)	0.0396 (6)	
C3	0.7287 (3)	0.4235 (3)	1.0597 (3)	0.0495 (7)	
H3	0.6371	0.4686	1.1067	0.059*	
C4	0.7911 (4)	0.2837 (3)	1.1216 (3)	0.0611 (8)	
H4	0.7402	0.2340	1.2097	0.073*	
C5	0.9294 (4)	0.2166 (3)	1.0533 (3)	0.0622 (8)	
H5	0.9717	0.1225	1.0965	0.075*	
C6	1.0048 (3)	0.2884 (3)	0.9218 (3)	0.0513 (7)	
H6	1.0975	0.2428	0.8765	0.062*	
C7	0.9425 (3)	0.4288 (3)	0.8570 (2)	0.0391 (6)	
C8	1.0286 (3)	0.5067 (3)	0.7161 (2)	0.0397 (6)	
C9	0.9476 (3)	0.6507 (3)	0.6424 (2)	0.0368 (6)	
C10	0.8044 (3)	0.7199 (3)	0.7110 (2)	0.0368 (6)	
C11	0.7303 (3)	0.8527 (3)	0.6356 (3)	0.0404 (6)	
C12	0.7934 (3)	0.9195 (3)	0.4973 (3)	0.0499 (7)	
H12	0.7422	1.0084	0.4494	0.060*	
C13	0.9328 (3)	0.8533 (3)	0.4309 (3)	0.0499 (7)	
H13	0.9762	0.8979	0.3381	0.060*	
C14	1.0076 (3)	0.7220 (3)	0.5014 (2)	0.0401 (6)	
C15	0.5922 (3)	1.0057 (3)	0.7771 (3)	0.0452 (6)	
C16	0.4227 (3)	1.0665 (3)	0.8308 (3)	0.0611 (8)	
H16A	0.4327	1.1330	0.8834	0.092*	
H16B	0.3629	0.9895	0.8918	0.092*	
H16C	0.3634	1.1162	0.7518	0.092*	
C17	1.2978 (3)	0.6659 (3)	0.4340 (3)	0.0429 (6)	
C18	1.4156 (3)	0.5881 (3)	0.3400 (3)	0.0576 (8)	
H18A	1.5278	0.6028	0.3427	0.086*	
H18B	1.3903	0.6249	0.2441	0.086*	
H18C	1.4044	0.4867	0.3726	0.086*	

### Atomic displacement parameters (Å^2^)

**Table:**

	*U*^11^	*U*^22^	*U*^33^	*U*^12^	*U*^13^	*U*^23^
O1	0.0493 (11)	0.0585 (13)	0.0589 (12)	0.0042 (9)	0.0220 (9)	−0.0221 (10)
O2	0.0333 (10)	0.0549 (12)	0.0680 (12)	0.0058 (9)	0.0123 (8)	−0.0209 (9)
O3	0.0339 (10)	0.0549 (12)	0.0566 (11)	0.0094 (8)	0.0009 (8)	−0.0273 (9)
O4	0.0466 (11)	0.0685 (14)	0.0596 (12)	−0.0058 (10)	0.0038 (9)	−0.0296 (10)
O5	0.0340 (10)	0.0652 (12)	0.0453 (10)	−0.0015 (8)	0.0077 (7)	−0.0302 (9)
O6	0.0452 (11)	0.0624 (13)	0.0653 (12)	−0.0046 (9)	−0.0026 (9)	−0.0298 (10)
C1	0.0283 (12)	0.0511 (16)	0.0434 (14)	−0.0036 (11)	0.0039 (10)	−0.0219 (12)
C2	0.0325 (13)	0.0483 (16)	0.0408 (14)	−0.0068 (11)	−0.0009 (10)	−0.0178 (11)
C3	0.0435 (15)	0.0585 (19)	0.0464 (15)	−0.0107 (13)	0.0052 (11)	−0.0182 (13)
C4	0.066 (2)	0.064 (2)	0.0482 (16)	−0.0156 (16)	0.0012 (14)	−0.0086 (14)
C5	0.065 (2)	0.0511 (19)	0.0637 (19)	−0.0023 (15)	−0.0097 (15)	−0.0069 (14)
C6	0.0441 (15)	0.0508 (18)	0.0564 (17)	0.0030 (13)	−0.0045 (12)	−0.0162 (14)
C7	0.0315 (13)	0.0452 (15)	0.0429 (14)	−0.0041 (11)	−0.0026 (10)	−0.0171 (11)
C8	0.0275 (12)	0.0465 (15)	0.0486 (14)	0.0001 (11)	−0.0018 (10)	−0.0226 (12)
C9	0.0274 (12)	0.0437 (15)	0.0425 (14)	−0.0022 (10)	0.0013 (10)	−0.0213 (11)
C10	0.0284 (12)	0.0454 (15)	0.0397 (13)	−0.0009 (11)	0.0013 (10)	−0.0215 (11)
C11	0.0289 (13)	0.0473 (16)	0.0471 (14)	0.0024 (11)	0.0023 (10)	−0.0240 (12)
C12	0.0510 (16)	0.0488 (16)	0.0455 (15)	0.0042 (13)	−0.0015 (12)	−0.0140 (12)
C13	0.0499 (16)	0.0553 (18)	0.0388 (14)	−0.0027 (13)	0.0054 (11)	−0.0125 (12)
C14	0.0287 (13)	0.0532 (17)	0.0416 (14)	−0.0038 (11)	0.0050 (10)	−0.0238 (12)
C15	0.0437 (16)	0.0456 (16)	0.0422 (14)	0.0037 (13)	0.0030 (11)	−0.0152 (12)
C16	0.0473 (17)	0.067 (2)	0.0661 (19)	0.0093 (15)	0.0079 (13)	−0.0304 (15)
C17	0.0333 (14)	0.0476 (16)	0.0444 (14)	0.0007 (12)	0.0014 (11)	−0.0137 (12)
C18	0.0386 (15)	0.071 (2)	0.0622 (18)	0.0051 (14)	0.0064 (12)	−0.0307 (15)

### Geometric parameters (Å, º)

**Table:**

O1—C1	1.226 (3)	C6—H6	0.9300
O2—C8	1.227 (3)	C7—C8	1.497 (3)
O3—C15	1.365 (3)	C8—C9	1.489 (3)
O3—C11	1.402 (3)	C9—C14	1.409 (3)
O4—C15	1.202 (3)	C9—C10	1.424 (3)
O5—C17	1.366 (3)	C10—C11	1.395 (3)
O5—C14	1.404 (3)	C11—C12	1.384 (4)
O6—C17	1.199 (3)	C12—C13	1.379 (3)
C1—C2	1.478 (4)	C12—H12	0.9300
C1—C10	1.504 (3)	C13—C14	1.371 (4)
C2—C3	1.401 (3)	C13—H13	0.9300
C2—C7	1.403 (3)	C15—C16	1.494 (3)
C3—C4	1.376 (4)	C16—H16A	0.9600
C3—H3	0.9300	C16—H16B	0.9600
C4—C5	1.386 (4)	C16—H16C	0.9600
C4—H4	0.9300	C17—C18	1.494 (3)
C5—C6	1.379 (4)	C18—H18A	0.9600
C5—H5	0.9300	C18—H18B	0.9600
C6—C7	1.387 (3)	C18—H18C	0.9600
			
C15—O3—C11	117.07 (19)	C9—C10—C1	119.7 (2)
C17—O5—C14	118.35 (18)	C12—C11—C10	121.9 (2)
O1—C1—C2	120.6 (2)	C12—C11—O3	116.2 (2)
O1—C1—C10	121.2 (2)	C10—C11—O3	121.8 (2)
C2—C1—C10	118.13 (19)	C13—C12—C11	119.5 (3)
C3—C2—C7	119.3 (2)	C13—C12—H12	120.3
C3—C2—C1	119.2 (2)	C11—C12—H12	120.3
C7—C2—C1	121.5 (2)	C14—C13—C12	120.1 (2)
C4—C3—C2	120.0 (2)	C14—C13—H13	119.9
C4—C3—H3	120.0	C12—C13—H13	119.9
C2—C3—H3	120.0	C13—C14—O5	116.3 (2)
C3—C4—C5	120.4 (3)	C13—C14—C9	122.1 (2)
C3—C4—H4	119.8	O5—C14—C9	121.3 (2)
C5—C4—H4	119.8	O4—C15—O3	122.9 (2)
C6—C5—C4	120.4 (3)	O4—C15—C16	126.7 (2)
C6—C5—H5	119.8	O3—C15—C16	110.3 (2)
C4—C5—H5	119.8	C15—C16—H16A	109.5
C5—C6—C7	120.0 (2)	C15—C16—H16B	109.5
C5—C6—H6	120.0	H16A—C16—H16B	109.5
C7—C6—H6	120.0	C15—C16—H16C	109.5
C6—C7—C2	119.9 (2)	H16A—C16—H16C	109.5
C6—C7—C8	119.3 (2)	H16B—C16—H16C	109.5
C2—C7—C8	120.7 (2)	O6—C17—O5	123.0 (2)
O2—C8—C9	122.9 (2)	O6—C17—C18	127.4 (2)
O2—C8—C7	119.4 (2)	O5—C17—C18	109.6 (2)
C9—C8—C7	117.76 (19)	C17—C18—H18A	109.5
C14—C9—C10	117.6 (2)	C17—C18—H18B	109.5
C14—C9—C8	121.31 (19)	H18A—C18—H18B	109.5
C10—C9—C8	121.1 (2)	C17—C18—H18C	109.5
C11—C10—C9	118.8 (2)	H18A—C18—H18C	109.5
C11—C10—C1	121.5 (2)	H18B—C18—H18C	109.5

### Hydrogen-bond geometry (Å, º)

**Table:**

*D*—H···*A*	*D*—H	H···*A*	*D*···*A*	*D*—H···*A*
C18—H18*A*···O2^i^	0.96	2.51	3.425 (4)	159

Symmetry code: (i) −*x*+3, −*y*+1, −*z*+1.
